# The Influence of the Partial Replacing of Inorganic Salts of Calcium, Zinc, Iron, and Copper with Amino Acid Complexes on Bone Development in Male Pheasants from Aviary Breeding

**DOI:** 10.3390/ani9050237

**Published:** 2019-05-13

**Authors:** Marian Flis, Dariusz Gugała, Siemowit Muszyński, Piotr Dobrowolski, Małgorzata Kwiecień, Eugeniusz R. Grela, Ewa Tomaszewska

**Affiliations:** 1 Department of Zoology and Animal Ecology, Faculty of Biology, Animal Sciences and Bioeconomy, University of Life Sciences in Lublin, Akademicka St. 13, 20-950 Lublin, Poland; m.flis@pzlow.pl (M.F.); gugaladariusz@o2.pl (D.G.); 2 Department of Biophysics, Faculty of Production Engineering, University of Life Sciences in Lublin, Akademicka St. 13, 20-950 Lublin, Poland; siemowit.muszynski@up.lublin.pl; 3 Department of Comparative Anatomy and Anthropology, Maria Curie-Sklodowska University, Akademicka St. 19, 20-033 Lublin, Poland; piotr.dobrowolski@umcs.lublin.pl; 4 Institute of Animal Nutrition and Bromatology, Faculty of Biology, Animal Sciences and Bioeconomy, University of Life Sciences in Lublin, Akademicka St. 12, 20-950 Lublin, Poland; malgorzata.kwiecien@up.lublin.pl (M.K.); ergrela@interia.pl (E.R.G.); 5 Department of Animal Physiology, Faculty of Veterinary Medicine, University of Life Sciences in Lublin, Akademicka St. 12, 20-950 Lublin, Poland

**Keywords:** mineral chelates, pheasants, bones, biomechanical endurance, nutrition

## Abstract

**Simple Summary:**

A significant problem of birds reared in farms and then reintroduced to natural living environment is their survival, which is usually much lower than that of the free-living ones. Behavioral and physiological deficiencies rather than morphological anatomy decide about failure, nevertheless, the birds’ body condition, including quality and maturity of skeletal system, are also important. In this context, the problem of proper nutrition for growing game birds is a major one. The level and source of microelements, as well as the source of protein are the main factors affecting bone growth in young captive-reared birds. Since calcium, zinc, iron, and copper are critical nutrients in all practical diets, this experiment was undertaken to determine the possibility of the partial inclusion of organic forms of these elements to the diet of pheasants in order to improve their bone development and survival.

**Abstract:**

This study analyzed the effects of partial replacing of Ca, Fe, Zn, and Cu salts with glycine chelates on the measures of bones health in 16-week-old captive-reared male pheasants, allocated to one of the three experimental groups supplemented with Ca, Fe, Zn, and Cu in forms of inorganic salts (the control group) or groups receiving from the ninth week 25% and 50% of supplemented elements as glycine chelates. At the end of rearing birds receiving chelates were heavier (*p* < 0.001) and their tibia showed an increase of numerous mechanical parameters: yield and ultimate force (*p* = 0.028, *p* < 0.001, respectively), stiffness (*p* = 0.007), Young modulus (*p* < 0.001), compared to the control animals. The bones of birds receiving chelates in 50% were also heavier (*p* < 0.001) and longer (*p* = 0.014), with thinner cortical bone in midshaft (*p* = 0.027) and thicker proximal trabeculae (*p* < 0.001) compared to the control. While both doses of chelates increased mineral density in midshaft (*p* = 0.040), bone content of Cu and Zn decreased (*p* = 0.025, *p* < 0.001, respectively). The content of immature collagen in cancellous bone and articular cartilage increased in groups receiving chelates (*p* < 0.001, *p* = 0.001, respectively). In conclusion, glycine chelates probably enhanced development of the skeletal system in male pheasants as bones were denser and more resistant to mechanical damage.

## 1. Introduction

The primary purpose of captive-reared pheasants in many European countries, including the United Kingdom and Poland, is their reintroduction to the natural living environment [1,2,3,4]. However, a significant problem of birds reared in farms and then reintroduced to natural living environment is their survival, which is usually much lower than 10% [5,6]. Behavioral and physiological deficiencies, rather than morphological anatomy decide about failure [4]. Nevertheless, the birds’ body condition, including quality and maturity of the keletal system, are also important [7,8].

Calcium is one of the main elements involved in skeletal mineralization and mechanical strength of bones [9,10]. Zinc is necessary for normal growth and bone development. In poultry, zinc deficiency results in reduction of weight gain, skeletal malformation and insufficient mineralization [11,12,13]. Iron is a component of a variety of enzymes involved in synthesis of collagen, the most abundant protein forming connective tissue and prevents osteoporosis [14,15,16]. Copper is essential for normal growth, bone development and metabolism [17,18,19]. Copper is needed for the action of lysyl oxidase, a Cu-dependent enzyme, which mediates the final step in the biosynthesis of collagen and normalizes the deposition of calcium and phosphorus in bones [20]

Nutrients recommendations for pheasants (per 1 kg of diet) are 5 mg for copper, 60 mg for zinc, 60 mg for iron, and 5.3 g for calcium [21]. Dietary minerals can be given in inorganic forms (as sulphates or carbonates) or chelated forms characterized with a higher bioavailability due to significantly higher absorption rates in the intestine compared with soluble inorganic salts [22] and the fact that additional chelation is not required at the brush border of the cell membrane [23,24]. Chelate feeding is a strategy used to increase the nutritional reserves of poultry [22,25]. Studies by El-Husseiny et al. [26] and Favero et al. [27], who evaluated partial replacement of inorganic sulphates of Zn and Cu with organic amino acid complexes in breeder hen and broiler diets, show that with simultaneous diet supplementation with inorganic and organic forms of Zn and Cu significantly improve numerous tibia quality indices (weight, mineralization, thickness, moment of inertia, and breaking strength). Moreover, our recent studies have shown that the use of Zn, Cu, and Fe in the form of amino acid complexes in poultry diet even at lower doses than recommended can enhance bone strength [28,29] and promote the development of hyaline cartilage enhancing proteoglicans content in articular cartilage which prevent destabilization of the collagen network [30,31].

In the available literature, we found no studies concerning the assessment of the development of the skeletal system and bone quality of pheasants from a breeding farm. As a tibia serves as the model bone in studies on the quality of the skeleton of captive-reared birds, the aim of the study was to determine the effect of partial replacing calcium, iron, zinc, and copper salts with glycine chelates on the tibia mineral composition, mechanical properties, and histomorphometry of trabecular bone in male pheasants originating from aviary breeding.

## 2. Materials and Methods

### 2.1. Ethical Approval

The experimental procedures used throughout this study were approved by the Local Ethics Committee on Animal Experimentation of University of Life Sciences in Lublin, Poland (Resolution No. 22/2016 of 13 May 2016).

### 2.2. Animals

The experiment consisted of 18 healthy male pheasants (*Phasianus colchicus*) obtained from the breeding flock of the experimental station and kept on a commercial breeding farm. Up to the end of week 8, the pheasants were kept in the rearing house and fed the commercial standard feeds: starter (1–4 weeks), grower (5–8 weeks) (Table 1). From the 9th week the birds were individually transferred to separate partially roofed aviaries and randomly allocated to one of the three experimental groups (*n* = 6 in each group), according to the partial replacement of inorganic salt with glycine chelates in finisher mineral-vitamin premix (Table 2). Each aviary was equipped with automatic nipple drinkers combined with an automatic feeders. Water and feed (finisher, Table 1) were individually provided *ad libitum*. All the diets, isonitrogenous, isoprotein, and isoenergetic, were formulated to meet or exceed the nutritional requirement [21]. All of the experimental birds were individually weighed at the start (at the end of 8th week), in the middle (at the end of 12th week), and the end of experiment (at the end of 16th week). The birds were mechanically stunned and slaughtered by cutting the carotid arteries at the age of 16 weeks. The procedure was conducted at a commercial facility under the veterinary control.

### 2.3. Experimental Feeds

Copper, zinc, iron and calcium formulated as salts and glycine chelates were introduced into the mineral-vitamin premix to finisher feed. The control group received Ca, Fe, Zn, and Cu in forms of inorganic salts, while in experimental groups 25% (the Ch-25 group) and 50% (the Ch-50 group) of supplemented elements were given as glycine chelates. The Glystar Forte glycine chelates (Arkop, Bukowno, Poland), containing 16% of Zn, 16% of Cu, 16% of Fe, and 20% of Ca, respectively, were used in the experiment. The amount of minerals in the premix was based on nutritional recommendations for pheasants, irrespective of its content in the components of the basal diet (Table 1). Recommendations for supplementation for pheasants are: 5 mg for copper, 60 mg for zinc, 60 mg for iron and 5.3 g for calcium (per 1 kg of diet) [21].

### 2.4. Bone Analysis

The weight and length of both isolated tibiae were measured, next they were frozen at −25 °C for further analyses. Bone metabolism was assessed by determining the bone mineral content and mineral density with the dual-energy X-ray absorptiometry method using a densitometer (Norland XR 43, Fort Atkinson, WI, USA). The measurements were performed for whole bone and separately for distal and proximal parts as well as for bone midshaft.

To estimate the mechanical properties of tibia mid-diaphysis a three-point bending test was performed using a universal testing machine (Zwick Z010, Zwick GmbH & Co. KG, Ulm, Germany). The load was applied with a displacement rate of 10 mm/min in the anterior-posterior plane (A-P plane) until the bone fracture [34,35]. During the test, the load-deformation curves were registered continuously. Next, the external and internal diameters of the mid-diaphysis cross-section were measured in horizontal (medial-lateral, M-L) and A-P planes with a digital caliper. On the basis of the measured diameters, the following geometric traits were calculated: cortical cross-sectional area, cortical index, mean relative wall thickness, and cross-sectional moment of inertia about medial-lateral axis [36,37]. On the basis of recorded load-deformation curves and calculated geometrical traits the mechanical properties of tibia were calculated using standard engineering beam-theory equations as described previously [36]. They included the determination of the values of yield force, ultimate force, stiffness, elastic energy, work to fracture, bending moment, Young modulus, yield strain, ultimate strain, yield stress, and ultimate stress.

### 2.5. Histomorphometry and Collagen Content Analysis

Samples of cartilage and bone were taken from the middle of the lateral tibial condyle. Sagittal 0.5 mm thick sections of cartilage and bone were cut perpendicular to the articular surface, next were formaldehyde-fixed, decalcified in EDTA solution and subjected to standard histological procedures [38]. Masson trichrome staining was used to assess the morphology of articular cartilage and Picrosirius red (PSR) staining was used to evaluate the distribution of thick and thin collagen fibers in articular cartilage, cancellous and trabecular bone [39]. All microscopic images were collected using an BX63 microscope and CellSens software (Olympus, Tokyo, Japan).

The bone volume (BV) and tissue volume (TV) were measured in the microscopic images of trabecular bone sections using the pixel count, and the relative bone volume (BV/TV) was assessed. Calculated morphometric traits included also mean trabecular thickness (Tb.Th), mean trabecular separation (Tb.Sp) and trabecular number (Tb.N). The trabecular bone morphometry was assessed using the ImageJ software (Wayne Rasband, NIMH, Bethesda, MD, USA).

### 2.6. Bone Ash and Macro- and Microelements Content

Bone ash determination was conducted by ashing moisture- and fat-free bones in 600 °C in muffle furnace. The cartilage caps were excluded in making the ash determinations. The mineral composition of bone was determined using ICP-MS spectrometry (Varian 820-MS, Palo Alto, CA, USA) in ashed bone samples. The macro- and microelements content in samples were expressed mg or µg in 1 g of crude ash.

### 2.7. Statistical Analysis

Each separately caged bird was considered as experimental unit, with 6 birds as 6 replications in each group. Statistical analyses were performed using a one-way analysis of variance (ANOVA) with Statistica 13.3 software (TIBCO Software Inc., Palo Alto, CA, USA). Differences at *p* < 0.05 were considered statistically significant. When significant differences were detected in the one-way ANOVA, a *post hoc* Tukey’s honest significant difference test was employed to compare differences among treatment means. Data are presented as means with pooled standard error of the mean (SEM).

## 3. Results

### 3.1. Rearing Results

The weight of birds at the beginning of experimental feeding (at the end of eighth week) did not differ between groups (Table 2). At the age of 12 weeks birds from the Ch-25 group weighed comparable to the control birds. Birds from the Ch-50 group were heavier compared to other groups and mean live weight of birds of Ch-50 treatment was significantly higher than that of control by 7% (67 g). At the end of the experiment (at the end of 16th week) birds from both groups supplemented with glycine chelates, irrespective of their amount, were heavier compared to the control animals, by 2.5% (26 g) and 3.5% (37 g), for the Ch-25 and Ch-50 groups, respectively.

### 3.2. Bone Morphology, Geometry, and Mechanical Traits

The replacement with glycine chelates in 50% resulted in heavier (by 12%, 0.75 g) and longer (by about 5%, 6 mm) tibia compared to the control group (Table 3). Moreover, the decrease of cortical index by about 8% (2.8%) was observed in birds supplemented with glycine chelates at the concentration of 50%. No effects of the treatments on the weight/length ratio, bone midshaft cross-sectional diameters and area, moment of inertia, mean relative wall thickness, and cortical index were noted.

The increase of the mean value of the yield force was noted in the group Ch-25 and Ch-50 by about 30% (29.4 N) and 28% (27.4 N) compared to the control group, respectively (Table 4). Similarly, the mean Young modulus increased by 34% (3.7 GPa) and 32% (3.5 GPa), stiffness by 29% (78 N/mm) and 21% (56 N/mm), and bending moment by 31% (0.33 Nm) and 34% (0.37 Nm) for the Ch-25 and Ch-50 group, respectively. Greater differences between control and experimental groups were observed for ultimate force (increase by 43% (56 N) and 56% (71 N) for the Ch-25 and Ch-50 group, respectively), and for work to fracture (increase by 60% (31 mJ) and 95% (48 mJ) for the Ch-25 and Ch-50 group, respectively). The increase of ultimate stress was observed only in the Ch-50 group when comparing to the control group (by 60%, 54.8 MPa). No effect of the treatments on elastic energy, yield strain, yield stress, and ultimate strain was noted (Table 4).

### 3.3. Bone Mineral Density, Mineral Content, and Ash Percentage

The administration of glycine chelates increased bone mineral density in the midshaft of tibia compared to the control group, on average by 51% (0.039 g/cm^2^) and 30% (0.023%) for Ch-25% and Ch-50% group, respectively (Table 5). Other changes were not observed.

### 3.4. Trabecular Histomorphometry, Immature Collagen Content

Glycine chelates, irrespective of their amount, did not influence relative bone volume in tibial trabecular bone (Table 6). The trabecular thickness increased in the Ch-50 group (by 14%, 7.7 μm, when comparing to the control) while the trabecular space decreased in the Ch-25 group compared to other groups (by 29%, 99 μm, when comparing to the control group). Trabecular number did not change in the Ch-25 group, while decreased in the Ch-50 group (by 20%, 0.09/mm, when comparing to the control).

The content of thin (immature) collagen in trabecular bone decreased in the Ch-50 group by 38% (6.8%) in the Ch-50 group (Table 7). However, in articular cartilage its content increased in birds from both groups supplemented with glycine chelates by 39% (4.1%) and 68% (7.17%) in the Ch-25 and Ch-50 group, respectively. In cancellous bone, the increase of immature collagen was also observed in both glycine chelates-supplemented groups. The highest content was observed in the Ch-50 group, where 2.7-fold increase was observed compared to the control group.

### 3.5. Bone Macro- and Microelements Content

The content of Cu and Zn decreased in both groups supplemented with glycine chelates (Table 8). Copper decreased by 23% (0.24 mg/kg) and 18% (0.19 mg/kg) in the Ch-25 and Ch-50 group, respectively. For both experimental groups, Zn decreased by 5% (14–17 mg/kg). No other changes in bone macro- and microelements content were observed.

## 4. Discussion

The problem of proper nutrition for captive-reared game birds is a major one and leg deformities in birds might result in a high rate of mortality after introduction to natural environment, where bones are exposed to extensive bending and torsion loads, tension, or compression [40,41,42]. The skeleton in wild birds needs to be lightweight to minimize the metabolic cost of flight, and strong enough to withstand the forces encountered during movement [8]. Therefore, special emphasis is placed on proper bone development which ensure proper movement and behavior. The level and source of microelements, as well as the source of protein are the major factors affecting bone growth in birds [13,28,43,44]. Bone formation is highly dependent on the dietary concentrations of calcium and phosphorus [45]. In birds, bone ash is a very important sensitive indicator of availability of minerals [46]. Furthermore, a bone ash percentage and the internal structure of tibiae are signs of rickets in pheasants which are very sensitive for changes in the diet [40].

Bone ash, Ca, and P content in bone as well as the Ca/P ratio in our pheasants did not differ between groups. However, tibiae in pheasant fed a diet with replacement of glycine chelates at the level of 50% were longer and heavier. The maturity of tibiae assessed on the basis of their diameters showed a tendency to higher horizontal external diameter in pheasants fed diet with glycine chelates irrespective of the level of replacement. Moreover, a tendency to larger both internal diameters of tibiae in birds fed diet with replacement of glycine chelates in 50% could indicate that the bone became more mature with greater marrow cavity. This is confirmed by other geometric measurements. With the same cross-sectional area, bone wall thickness (cortical index) in the Ch-50 group was the lowest. It is important and should be emphasized that between two bones with the same cross-sectional area loaded with the same load, smaller deflection for a bone with larger diameters will be observed. In turn, to get the same deflection for large and low-diameters bones, higher force is needed for a large-diameters bone. In our study, the bone deflection (in terms of ultimate strain) was the same in all groups, the observed ultimate forces were the highest in the Ch-50 group.

Bird bones are characterized by a much thinner cortical bone, compared to terrestrial animals. The bending strength and Young modulus are significantly higher for marrow-filled than pneumatic bones in birds. Also, the ratio of internal to external diameter is larger in pneumatic bones than in marrow filled bones [47]. With the same cross-sectional area, the bone with larger diameters is more mechanically resistant [48]. In general, birds’ bones are denser and stronger relative to their weight. For a bone of a given volume and length, bone stiffness and strength can be maintained via a trade-off between bone density and shape [8]. Bone density reflects its mineral content and is highly correlated with bone rigidity (Young modulus, the ability to resist deformation) and strength (ultimate stress, the ability to resist fracture). Additionally, even a small bone can be strong and stiff if it is composed of dense bone tissue [49].

In our study, not only bones with larger bone marrow cavity (the Ch-50 group) had higher bone mineral density in midshaft, but also in the group fed diet with glycine chelates in lower amount (the Ch-25 group) bone mineral density was higher compared to the control group. This increased mineral density resulted in stronger bones of pheasants from these groups, as shown by higher values of mechanical traits (ultimate force, Young modulus, work to fracture) assessed in three-point bending test.

Pheasant chicks are especially prone to leg disorders and abnormal development when a certain key nutrient, such as zinc, is inadequate [50,51,52,53,54]. Long bones of the legs and wings are shorter and thicker than normal [52,55]. Similar effects are observed in copper and zinc deficiency [56,57]. Iron also participates in a numerous enzymatic systems involved in collagen synthesis and participates in bone metabolism through vitamin D activation and deactivation [15,58]. Calcium and phosphorus, in increasing amounts in the diet, exert independent, adverse effects on iron utilization [59].

Our pheasant fed diets with glycine chelates had decreased content of copper and zinc in bone tissue. Additionally, the increase of immature collagen in cancellous bone was observed in relation to the amount of glycine chelates in the feed. The presence of immature collagen may indicate an intense bone remodeling, but also unfinished growth in volume. On the other hand, higher content of mature collagen in trabecular bone in the Ch-50 group was noted. This shows how bone diaphysis differs from trabecular bone, which is characterized by a different proportion of the organic and inorganic phases that compact bone. The presence of immature (thin) collagen in the articular cartilage observed in glycine chelates supplemented groups (depending on the amount of their inclusion) once again indicates that the bone turnover process is more intensive. The higher level of glycine chelates increased a mature collagen content in trabecular bone and improved histomorphological microarchitecture of trabecular bone compared to control and Ch-25 groups by increasing the thickness of trabeculae. However, the reduction of the trabecular number was noted and no changes in bone real volume was observed.

Moreover, body weight gained by our pheasants supplemented with glycine chelates, while being greater than in the control, still represents only approximately 90% of the weight of an adult pheasant in natural hunting grounds [60]. This may be one of the reasons why farmed birds have a lower chance to survive after introduction to natural environment. The improvement of the quality of skeleton might be one of the of way increasing birds’ survival rate after release. Thus, future research should also cover the measurements of post-release survival rate of tagged birds.

## 5. Conclusions

The partial replacement of inorganic salts by Ca, Cu, Fe, and Zn glycine chelates in finisher feed of captive-reared male pheasants resulted in bones with the greater ability to resist deformation and fracture. Taken together, the results obtained strengthen the hypothesis that chelates provided in to the birds diet, including pheasants, promotes bone development. These results also provide a strong evidence for the need of selection of feed supplements if we want to maximize the chances of pheasants to survive upon the release to natural environment.

## Figures and Tables

**Table 1:** Composition and nutritive value of reared pheasant diet.

	Starter (1–4 Weeks)	Grower (5–8 Weeks)	Finisher (9–16 Weeks)
Ingredients, g/kg			
Corn	232.1	272.3	291.6
Wheat	100.0	100.0	160.0
Soybean meal	280.0	280.0	250.0
Garden pea	50.0	50.0	50.0
Fish meal	80.0	20.0	
Linseed	40.0	40.0	40.0
Sunflower meal	80.0	80.0	80.0
Soya oil	50.0	50.0	
Sorghum	10.0	30.0	50.0
Dicalcium phosphate	16.0	16.0	17.0
Calcium carbonate	55.0	55.0	55.0
Salt	3.0	3.0	3.0
Mineral-vitamin premix ^1^	2.5	2.5	2.5
DL‒methionine	0.5	0.4	0.3
L‒lysine chloride	0.9	0.8	0.6
Calculated composition			
AMEn, MJ/kg ^2^	12.05	12.06	10.57
Analyzed composition ^3^			
Dry matter g/kg	896.9	895.4	894.8
Crude protein, g/kg	278.7	230.7	189.2
Crude ash, g/kg	70,1	69,2	69,3
Calcium, g/kg	27.1	26.9	26.4
Total phosphorus, g/kg	7.9	7.8	7.7
Iron, mg/kg	179.2	176.3	174.5
Zinc, mg/kg	132.4	129.5	125.4
Copper, mg/kg	22.9	22.8	22.7

^1^ The mineral-vitamin premix provided in 1 kg diet: Mn 60 mg, I 1 mg, Fe 54 mg, Zn 100 mg, Cu 11 mg, Se 0.2 mg, vitamin A 10,000 IU, vitamin D3 2500 IU, vitamin E 50 mg, vitamin K3 2 mg, vitamin B1 1.5 mg, vitamin B2 4.5 mg, vitamin B6 3 mg, vitamin B12 0.015 mg, biotin 0.1 mg, folic acid 0.8 mg, nicotinic acid 20 mg, pantothenic acid 12 mg, choline 300 mg.^2^ AMEn – metabolizable energy at zero nitrogen balance calculated with Fisher and McNab equations [32].^3^ The chemical composition of the basal diets and mineral content in the feed samples was analyzed according to AOAC procedures [33].

**Table 2:** Body weights (g) of male pheasants.

Birds Age	The Control ^1^	The Ch-25 Group ^1^	The Ch-50 Group ^1^	SEM ^2^	*p*-Value
8 weeks	492	495	496	2	0.262
12 weeks	891 ^a^	903 ^a^	958 ^b^	3	<0.001
16 weeks	1049 ^a^	1075 ^b^	1086 ^b^	4	<0.001

^1^ Control: supplemented with Ca, Fe, Zn, and Cu in forms of inorganic salts; Ch-25: 25% of supplemented elements were given in the form of glycine chelates; Ch-50: 50% of supplemented elements were given in the form of glycine chelates; Values are presented as mean (*n* = 6). ^2^ SEM: pooled standard error of the mean. ^a,b^ Mean values within a row with different superscripts differ significantly at *p* < 0.05 (Tukey test).

**Table 3:** Weight and geometrical characteristics of tibia of 16-week-old male pheasants.

Dependent Variable	The Control ^1^	The Ch-25 Group ^1^	The Ch-50 Group ^1^	SEM ^2^	*p*-Value
Bone weight, g	6.45 ^a^	6.79 ^a^	7.20 ^b^	0.28	<0.001
Bone length, mm	108.4 ^a^	109.7 ^ab^	114.4 ^b^	1.32	0.014
Bone weight/length ratio	5.96	6.35	6.30	0.27	0.546
M-L plane external diameter, mm	6.79	7.07	7.02	0.31	0.798
M-L plane internal diameter, mm	4.20	4.15	4.75	0.13	0.352
A-P plane external diameter, mm	5.56	5.37	5.55	0.13	0.477
A-P plane internal diameter, mm	3.62	3.59	3.68	0.10	0.839
Mean relative wall thickness	0.84	0.86	0.77	0.04	0.327
Cortical index, %	36.26 ^b^	37.13 ^b^	33.44 ^a^	1.47	0.027
Cross-sectional area, mm^2^	17.85	18.09	17.04	1.23	0.821
Moment of inertia, mm^4^	48.78	44.03	49.20	5.28	0.748

^1^ Control: supplemented with Ca, Fe, Zn, and Cu in forms of inorganic salts; Ch-25: 25% of supplemented elements were given in the form of glycine chelates; Ch-50: 50% of supplemented elements were given in the form of glycine chelates; Values are presented as mean (*n* = 6). ^2^ SEM: pooled standard error of the mean. ^a,b^ Mean values within a row with different superscripts differ significantly at *p* < 0.05 (Tukey test).

**Table 4:** Mechanical characteristics of tibia of 16-week-old male pheasants.

Dependent Variable	The Control ^1^	The Ch-25 Group ^1^	The Ch-50 Group ^1^	SEM ^2^	*p*-Value
Yield force, N	99.5 ^a^	128.9 ^b^	126.9 ^b^	7.7	0.028
Elastic energy, mJ	18.75	25.65	27.38	2.73	0.092
Ultimate force, N	128.3 ^a^	184.1 ^b^	199.7 ^b^	5.2	<0.001
Work to fracture, mJ	50.75 ^a^	81.40 ^b^	99.13 ^b^	8.04	0.003
Stiffness, N/mm	267.7 ^a^	346.5 ^b^	324.0 ^b^	15.2	0.007
Young modulus, GPa	10.8 ^a^	14.5 ^b^	14.3 ^b^	1.2	<0.001
Bending moment, N⋅m	1.08 ^a^	1.41 ^b^	1.45 ^b^	0.09	0.015
Yield strain, %	0.61	0.60	0.61	0.04	0.993
Yield stress, MPa	64.52	87.21	86.78	7.63	0.086
Ultimate strain, %	1.11	1.20	1.36	0.12	0.331
Ultimate stress, MPa	84.38 ^a^	123.86 ^ab^	139.16 ^b^	10.92	0.008

^1^ Control: supplemented with Ca, Fe, Zn, and Cu in forms of inorganic salts; Ch-25: 25% of supplemented elements were given in the form of glycine chelates; Ch-50: 50% of supplemented elements were given in the form of glycine chelates; Values are presented as mean (*n* = 6). ^2^ SEM: pooled standard error of the mean. ^a,b^ Mean values within a row with different superscripts differ significantly at *p* < 0.05 (Tukey test).

**Table 5:** Densitometric parameters of tibia of 16-week-old male pheasants.

Dependent Variable	The Control ^1^	The Ch-25 Group ^1^	The Ch-50 Group ^1^	SEM ^2^	*p*-Value
Bone mineral content, g					
Total bone	0.343	0.487	0.367	0.066	0.281
Bone midshaft	0.093	0.160	0.125	0.026	0.211
Bone distal part	0.167	0.193	0.112	0.030	0.191
Bone proximal part	0.097	0.110	0.150	0.030	0.457
Bone mineral density, g/cm^2^					
Total bone	0.098	0.112	0.096	0.006	0.182
Bone midshaft	0.076 ^a^	0.115 ^b^	0.099 ^b^	0.010	0.040
Bone distal part	0.111	0.114	0.101	0.009	0.552
Bone proximal part	0.099	0.111	0.107	0.006	0.413
Ash, %	48.5	48.2	48.1	0.2	0.733

^1^ Control: supplemented with Ca, Fe, Zn, and Cu in forms of inorganic salts; Ch-25: 25% of supplemented elements were given in the form of glycine chelates; Ch-50: 50% of supplemented elements were given in the form of glycine chelates; Values are presented as mean (*n* = 6). ^2^ SEM: pooled standard error of the mean. ^a,b^ Mean values within a row with different superscripts differ significantly at *p* < 0.05 (Tukey test).

**Table 6:** Trabecular bone morphology of tibia of 16-week-old male pheasants.

Dependent Variable	The Control ^1^	The Ch-25 Group ^1^	The Ch-50 Group ^1^	SEM ^2^	*p*-Value
Relative bone volume (BV/TV), %	24.68	22.06	22.97	0.88	0.260
Trabecular thickness (Tb.Th), μm	54.72 ^a^	48.21 ^a^	62.43 ^b^	2.00	<0.001
Trabecular space (Tb.Sp), μm	338 ^b^	239 ^a^	350 ^b^	9	<0.001
Trabecular number (Tb.N), 1/mm	0.46 ^a^	0.47 ^a^	0.37^b^	0.01	<0.001

^1^ Control: supplemented with Ca, Fe, Zn, and Cu in forms of inorganic salts; Ch-25: 25% of supplemented elements were given in the form of glycine chelates; Ch-50: 50% of supplemented elements were given in the form of glycine chelates; Values are presented as mean (*n* = 6). ^2^ SEM: pooled standard error of the mean. ^a,b^ Mean values within a row with different superscripts differ significantly at *p* < 0.05 (Tukey test).

**Table 7:** The content (%) of immature collagen in tibia of 16-week-old male pheasants.

Bone Tissue	The Control ^1^	The Ch-25 Group ^1^	The Ch-50 Group ^1^	SEM ^2^	*p*-Value
Trabeculae	17.56 ^a^	16.34 ^a^	10.89 ^b^	0.84	0.004
Articular cartilage	10.46 ^a^	14.58 ^b^	17.63 ^b^	0.70	0.001
Cancellous bone	9.42 ^a^	13.13 ^b^	25.42 ^c^	0.64	<0.001

^1^ Control: supplemented with Ca, Fe, Zn, and Cu in forms of inorganic salts; Ch-25: 25% of supplemented elements were given in the form of glycine chelates; Ch-50: 50% of supplemented elements were given in the form of glycine chelates; Values are presented as mean (*n* = 6). ^2^ SEM: pooled standard error of the mean. ^a,b,c^ Mean values within a row with different superscripts differ significantly at *p* < 0.05 (Tukey test).

**Table 8:** Bone macro- and microelements content in tibia of 16-week-old male pheasants.

Dependent Variable	The Control ^1^	The Ch-25 Group ^1^	The Ch-50 Group ^1^	SEM ^2^	*p*-Value
Ca, g/kg	372	371	365	2	0.059
Cu, mg/kg	1.04 ^b^	0.80 ^a^	0.85 ^a^	0.08	0.025
Fe, mg/kg	46.0	43.1	43.2	5.6	0.877
P, g/kg	145	144	145	1	0.170
Zn, mg/kg	318 ^b^	301 ^a^	304 ^a^	5	<0.001
Ca/P	2.56	2.58	2.53	0.01	0.189

^1^ Control: supplemented with Ca, Fe, Zn, and Cu in forms of inorganic salts; Ch-25: 25% of supplemented elements were given in the form of glycine chelates; Ch-50: 50% of supplemented elements were given in the form of glycine chelates; Values are presented as mean (*n* = 6). ^2^ SEM: pooled standard error of the mean. ^a,b^ Mean values within a row with different superscripts differ significantly at *p* < 0.05 (Tukey test).
